# The Team to Address Bariatric Care in Canadian Children (Team ABC3): Team Grant Research Proposal

**DOI:** 10.1186/s13104-017-2506-z

**Published:** 2017-10-05

**Authors:** Team ABC3, Laura N. Anderson, Geoff D. C. Ball, Catherine S. Birken, Annick Buchholz, Sarah Carsley, Jean-Pierre Chanoine, Hayyah Clairman, Elizabeth Dettmer, Mary Forhan, Nicole D. Gehring, Stasia Hadjiyannakis, Jill Hamilton, Rhona Hanning, Jess Haines, Josephine Ho, Nicholas L. Holt, Theresa H. M. Kim, Laurent Legault, Paola Luca, Jonathon L. Maguire, Katerina Maximova, Amy C. McPherson, Katherine M. Morrison, Louise C. Mâsse, Patricia C. Parkin, Arnaldo Perez, Ann E. Sprague, Alene Toulany, Mark S. Tremblay, Karen Tu

**Affiliations:** Department of Pediatrics, University of Alberta, 4-515 Edmonton Clinic Health Academy, 11407-87th Ave, Edmonton, AB T5K 0L4 Canada

**Keywords:** Severe obesity, Pediatrics, Epidemiology, Interventions, Health services research, Canada

## Abstract

**Background:**

Severe obesity (SO) in Canadian children remains poorly understood. However, based on international data, the prevalence of SO appears to be increasing and is associated with a number of psychosocial, bio-mechanical, and cardiometabolic health risks. The purpose of our national Team to Address Bariatric Care in Canadian Children (Team ABC3) is to develop and lead a series of inter-related studies to enhance the understanding and management of SO in Canadian children and adolescents (0–18 years).

**Methods/design:**

From 2015 to 2019, Team ABC3 will conduct a series of projects at the regional, provincial, and national levels using multiple methods and study designs to respond to key knowledge gaps by (i) generating evidence on the prevalence of SO and its impact on health services utilization in children using existing Canadian data sources from primary care settings, (ii) exploring contemporary definitions of SO that link with health outcomes, (iii) comparing and contrasting health risks across the continuum of SO, (iv) understanding potential barriers to and facilitators of treatment success in children with SO, and (v) examining innovative lifestyle and behavioral interventions designed to successfully manage SO in children and their families. Furthermore, to examine the impact of innovative interventions on the management SO, we will (vi) evaluate whether adding a health coach, who provides support via text, email, and/or phone, improves children’s ability to adhere to a web-based weight management program and (vii) test the feasibility and impact of a community-based weight management program for pre-school children with SO and their parents that combines group-based parenting sessions with in-home visits.

**Discussion:**

Our research aligns with national priorities in obesity research, brings together leading scientists, clinicians, and stakeholders from across Canada, and will inform health services delivery throughout the country to provide the best care possible for children with SO and their families.

## Background

In Canada, approximately one in three children is classified as either overweight or obese, but no national-level data are available to quantify the magnitude of severe obesity (SO ≥99th percentile of BMI) [1]. By way of comparison, in the United States, data suggest that the total proportion of children with obesity might have plateaued [2–4], but boys and girls with obesity have become even more obese over the past 15 years. As of 2014, ~6% of American children have SO [5], a proportion that is expected to increase to 18.4% by the year 2030 [6]. If similar trends hold true in Canada, there is a clear imperative for us to better understand the magnitude and impact of SO in children and their families.

Substantial heterogeneity exists in how SO is defined in children [5, 7–15]. In our experience, many Canadian pediatric weight management clinics use BMI ≥99th percentile to define SO, based on either the Centers for Disease Control and Prevention [16] or the World Health Organization [17] criteria. However, recent reports suggest that new definitions (e.g., weight ≥120% of the 95th percentile weight value) may provide increased specificity and improved ability to monitor changes over time [18, 19]. At this point in time, an evidence-based definition of SO is needed to reflect obesity-related health risks across the continuum of excess weight in children [19].

Several reports have identified health risks associated with SO in children, with many focusing on risk factors for type 2 diabetes and cardiovascular disease [8, 20, 21]. Other studies have linked SO in children with physical and cognitive disabilities [18], poor psychosocial and mental health [22–25], and detrimental health behaviors [26, 27]. Systematic reviews [28–33] have shown that most outpatient lifestyle and behavioral interventions for obesity management in children have a positive (albeit modest) impact on weight loss. Studies of “real-life” clinics show younger children and those with lower levels of obesity tend to achieve more clinically-meaningful weight loss while weight stabilization or maintenance appears to be a more realistic goal for older children and those with higher levels of obesity [34–36]. Improvements in obesity status are equally modest when lifestyle and behavioral interventions are supplemented by pharmacotherapy [37, 38]. Increasing evidence supports a role for bariatric surgery in managing SO, with substantial weight loss accompanied by improved cardiometabolic and psychosocial indicators [39], but the availability and long term evidence of pediatric surgical interventions is limited [40]. Collectively, these findings highlight the importance of novel, accessible, and effective interventions for managing SO and associated health risks.

The causes and consequences of SO are complex and necessitate applied clinical research that is diverse in scope and includes the application of inter-professional expertise. Consensus on effective clinical approaches to preventing and managing SO is lacking, at least in part due to limited evidence on which to base decision-making. The Team to Address Bariatric Care in Canadian Children (Team ABC3) was designed to respond to key knowledge gaps by (i) generating evidence on the prevalence of SO and its impact on health services utilization in children using existing Canadian data sources from primary care settings, (ii) exploring contemporary definitions of SO that link with health outcomes, (iii) comparing and contrasting health risks across the continuum of SO, (iv) understanding potential barriers to and facilitators of treatment success in children with SO, and (v) examining innovative lifestyle and behavioral interventions designed to successfully manage SO in children and their families. Furthermore, to examine the impact of innovative interventions on the management SO, we will (vi) evaluate whether adding a health coach, who provides support via text, email, and/or phone, improves children’s ability to adhere to a web-based weight management program and (vii) test the feasibility and impact of a community-based weight management program for pre-school children with SO and their parents that combines group-based parenting sessions with in-home visits.

## Methods/design

### Overview

Our research plan is to conduct a series of seven studies over a 5-year period to better understand and manage SO in children. The studies are diverse in setting (e.g., community, primary- and tertiary-level care) and methods (e.g., observational studies, clinical trials, and qualitative). Studies one through five, which address issues such as health risks and drivers of unhealthy weight gain, aim to better understand issues unique to children with SO, while studies six and seven aim to examine different intervention models for managing SO.

#### Study 1: Prevalence and health care utilization of SO in children accessing primary health care in Ontario

### Rationale and objectives

No national or provincial estimates of excess weight in Canadian children are available. This knowledge gap led to provincial recommendations for a system to monitor prevalence of childhood obesity through existing mechanisms, including primary care electronic medical records [18]. Children in Ontario attend ~19 primary health care visits in the first two years of life [41]. As it is standard care to measure height and weight at these visits [42], an excellent source of weight-related data should be available but to date has not been accessed. It has been suggested that health care utilization is higher among children with obesity versus their leaner peers [43–47]. Knowing the prevalence of SO and health care utilization of children with SO is essential to develop interventions, evaluate the impact of these interventions, and monitor trends over time. Thus, Team ABC3 objectives for this study are to determine (i) the prevalence of SO in three cohorts of 0 to 18 year olds in Ontario and (ii) whether SO is associated with increased all-cause health care utilization in 0–18 year olds.

### Research plan

Three Ontario-based data sources will be accessed: (1) The Applied Research Group for Kids (TARGet Kids!) [48], a practice-based research network in primary care that includes >7500 children, recruited between 0 to 5 years of age, with measured height and weight data, and obesity-related health behaviors and outcomes, (2) the Electronic Medical Record Administrative Data Linked Database (EMERALD) [49] which includes data collected during primary care visits of >30,000 children 0 to 18 years of age and (3) the Better Outcomes Registry and Network (BORN Ontario) [50] which collects, interprets, shares and protects health-related data about pregnancy, birth and childhood from primary care practices in Ontario. For objective 1, we will complete cross-sectional analyses to determine the prevalence of SO in the three data sources. Height/length, weight, age, sex, family income and postal code data will be extracted. Although BMI ≥99th percentile is our working definition of SO in children [17], we will also calculate prevalence by additional criteria (e.g., ≥120% of the 95th percentile) to create a comparative approach as done previously by our team members to determine obesity prevalence [51, 52]. Additionally, longitudinal analyses will be performed over different time periods based on available data from these datasets. Longitudinal data is available for children in TARGet Kids! from 2008 to present, and from EMERALD from 1997 to 2016. Two analytic approaches will be conducted: (1) following a subset of the same children over time and (2) using serial-cross-sectional prevalence estimates. For objective 2, we will undertake analyses to assess health care utilization of children with SO. We will link our three databases with Ontario’s administrative health services data housed at the Institute of Clinical Evaluative Sciences (ICES), through children’s Ontario Health Insurance Plan (OHIP) numbers. All-cause health care utilization will be defined as the number of hospitalizations, emergency department (ED) visits, and physician visits, including primary care and specialist visits. Using ICES databases, we will access the Canadian Institute of Health Information—Discharge Abstract Database, the National Ambulatory Care Reporting System (ED visits) and the OHIP billing claims database (physician visits). Descriptive statistics will be calculated for all variables to determine distributions. A multivariable Poisson regression will be performed to determine the association between SO and health care utilization adjusted for age, sex, family income, and geographic region (postal code). Sensitivity analyses will be used to determine how changes in the definition of SO affect the association with health care utilization. The primary analyses will be using the Canadian definition, prosed by the Dietitians of Canada, suggesting zBMI > 3 or ≥99.9th BMI percentile. We will also use the proposed definition of ≥120% of 95th percentile [53].

### Outcomes

This study will identify the prevalence of SO in children and the impact of SO on health care utilization in Ontario and demonstrate the feasibility of using primary care data to monitor SO prevalence and health care utilization. These findings have the potential to be extended to other provinces through our team of researchers and decision-makers.

#### Study 2: Predictors of treatment initiation for children with SO referred for tertiary-level management of obesity

### Rationale and objective

To benefit from lifestyle and behavioral interventions for managing SO, families must initiate treatment. Data are limited, but one recent study found that only 10–15% of children referred for additional care actually initiated obesity management services [54]. To optimize the impact of health services for managing SO in children, clinicians and health care administrators must understand the factors that influence treatment initiation in families. With that understanding, clinicians can make appropriate referrals and incorporate strategies to increase the likelihood that families engage in treatment. Therefore, we will examine demographic (children’s sex and age), anthropometric (children’s BMI z-score), procedural (type of referral provider, length of the enrollment process, and treatment clinic), and contextual (distance between families’ home and treatment venues and seasonality) variables possibly associated with initiation of multidisciplinary management for SO in children in the province of Alberta.

### Research plan

This retrospective study will examine data from all children (n ≈ 2500) referred by Alberta-based physicians and nurse practitioners to tertiary-level, multidisciplinary obesity management clinics (2005–2013). Since 2005, Alberta Health Services (AHS) has dedicated administrative and informatics infrastructure to receive and process referrals for pediatric obesity management, including a standard referral form and data management resources to convert referral data to electronic format. Team members helped to design, implement and refine the referral form and associated procedures. We will work with our AHS decision-maker partners to obtain institutional approval and access to demographic, geographic, anthropometric and clinical data, as well as history of obesity management and potential barriers to treatment, from children’s referral documents. Along with defining SO in children as BMI ≥99th percentile we will also calculate prevalence by additional criteria (e.g., ≥120% of the 95th percentile) to understand a more contemporary view of SO in children. Research by team members [55, 56] indicates that 50–90% of children referred for obesity management will have SO. We will perform descriptive statistics (e.g., means, 95% CIs, proportions) and multivariable logistic regression analyses, with treatment initiation as the dependent variable, to determine the proportion of children who initiate treatment and characteristics of families participating/not participating in care. Independent variables, both continuous and categorical, will be considered for inclusion in our models based on preliminary analyses, an approach used previously by our team members [57, 58]. Similar to previous studies [59], data will be managed using LabKey^®^, an open-source, password-protected data repository available to our team through the University of Alberta Women and Children’s Health Research Institute. We will not examine reasons, barriers, and facilitators of initiation, as our research team has previously investigated these factors [60, 61].

### Outcomes

This project will determine whether treatment initiation is related to SO and whether demographic, anthropocentric, procedural, and contextual factors are associated with family initiation of treatment, informing clinical or administrative strategies to help children and families overcome barriers to care.

#### Study 3: Pathways to eating: determining eating behavior phenotypes in children with SO

### Rationale and objective

Pediatric obesity is a complex condition with heterogeneous phenotypes, yet recommended treatments fail to target these multifaceted etiological pathways. Overeating is a major contributor to obesity; recent evidence supports notable differences in eating behaviors between children with and without obesity [62–66]. Various eating behaviors, including loss of control eating, emotional eating, excessive hunger, impulsivity/delay of gratification, and responsivity to external cues mark important and distinct triggers. They often co-occur [67] but are infrequently investigated, particularly in children with SO [67, 68]. We will determine eating behavior phenotypes in children with SO by examining the clustering of eating triggers, relating identified phenotypes to demographic, physical, and environmental characteristics of children with SO, and investigate whether treatment outcomes vary according to eating phenotypes and changes in eating pathways.

### Research plan

**Fig. 1:**
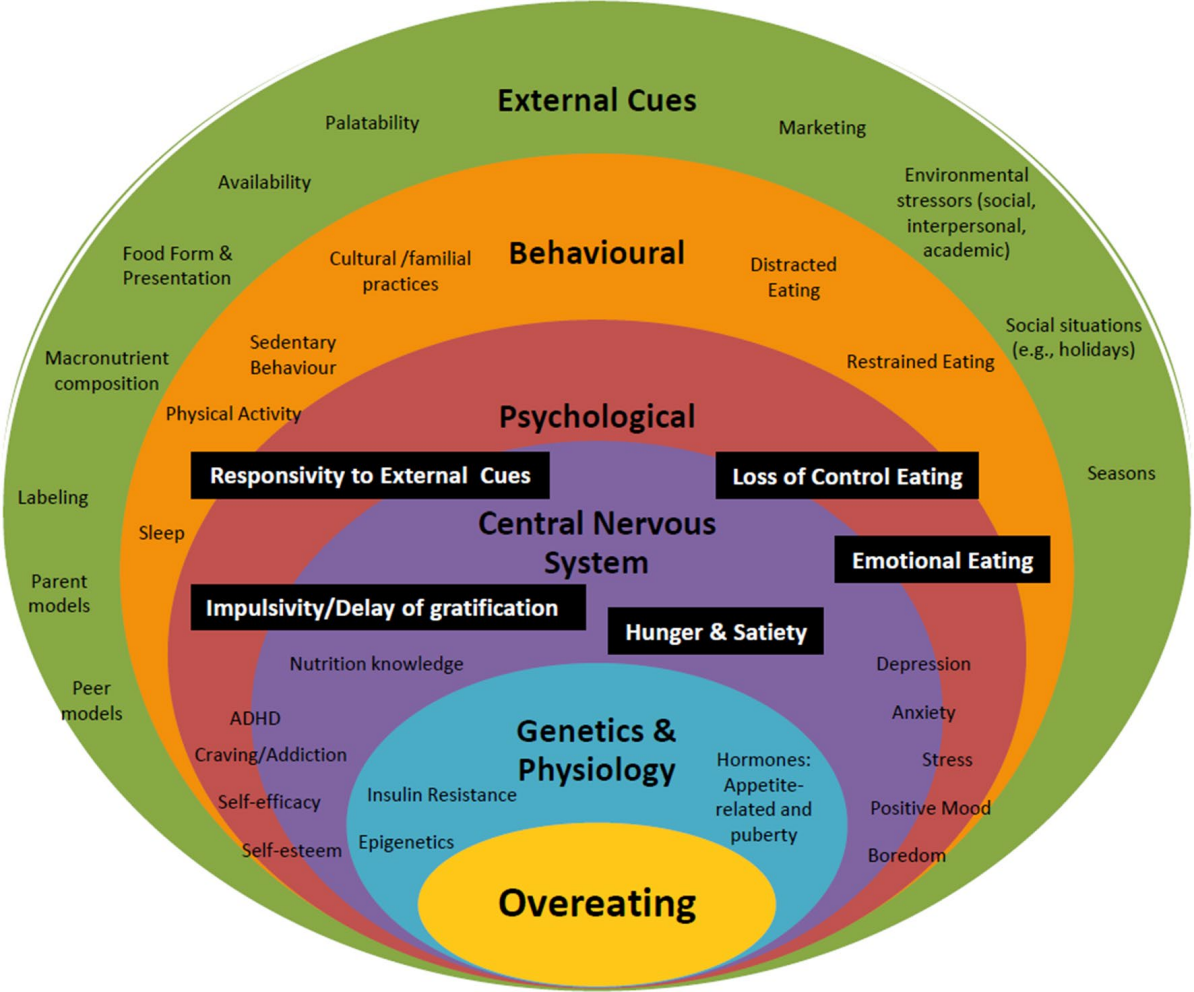
A model of pathways to eating in children with SO

The Canadian Pediatric Weight Management Registry (CANPWR) [55] is the largest obesity research study in Canada, encompassing ten multidisciplinary clinical centers. Through the CANPWR data collection process, we will recruit a sample of youth 10–18 years old (n = 500) for a longitudinal (2-year) sub-study. They will complete validated surveys (duration: ~15 min) during their annual CANPWR visits. Surveys will supplement existing demographic, anthropometric and lifestyle measures already being collected [55] and provide detailed information on five proposed key pathways: loss of control eating, emotional eating, hunger, impulsivity, and eating in response to external cues (Fig. 1). Data will be collected at three time points (0-, 12-, and 24-months follow-up). Eating phenotypes will be assigned membership in a latent cluster from patterns of interrelationships among indicator variables, using Latent Profile Analysis (LPA). LPA uses categorical and continuous indicators from cross-sectional data to identify latent subgroups of individuals (e.g., two to five mutually exclusive groups); a statistical approach our team members have applied previously [69, 70]. Second, we will compare demographic (e.g., age, sex) anthropometric (e.g., BMI), and environmental (e.g. family structure) variables across the identified LPA phenotypes using one-way analysis of variance and post-hoc comparisons. Third, we will examine treatment outcomes (e.g., longitudinal changes in demographic, physical, and environmental variables at 12- and 24-months follow-up) in relation to the LPA phenotypes and changes in individual eating components (e.g., improvements in impulse control), using mixed effects models.

### Outcomes

This research will reveal the degree to which triggers of eating are present in children with SO enrolled in CANPWR and offer insights into which specific triggers influence overeating, informing tailored interventions to improve eating behaviors.

#### Study 4: Family recommendations for improving health services to manage SO in children with disabilities

### Rationale and objectives

Low treatment initiation, high program attrition, and poor adherence to lifestyle and behavioral recommendations limit the successful management of pediatric obesity [54, 71–73]. Team members have explored family preferences for care which suggested that families desire better help from health care professionals, family-centered treatment, a desire for increased social support, and need for policy/program-level changes to assist their weight management efforts [74]. Extending this research, team members are currently completing a qualitative study exploring families’ reasons and decisions for initiating, terminating or continuing health care to manage pediatric obesity [59]. To date, we have interviewed four families who have a child with SO and a disability and identified several unique issues associated with SO in children with disabilities. Recent reviews note that children with disabilities are at a heightened risk of developing obesity [75, 76], but little information exists on managing obesity in children with any disabilities. Thus, we aim to explore families’ experiences in managing SO, and identify families’ recommendations for improving health services to manage SO in children with disabilities and their parents.

### Research plan

Parents (primary caregivers or guardians) and children with disabilities will be recruited through CANPWR sites in six of the largest cities across Canada (Vancouver, Edmonton, Hamilton, Toronto, Ottawa and Montreal) and through the pediatric outpatient clinic at a children’s rehabilitation centre in Toronto. We will enroll 35 parent–child dyads (n = 5 dyads per site) for a total of 70 interviews. English-speaking families will be eligible if children are 10–17 years old, have SO (BMI ≥99th percentile), and experience participation restrictions or activity limitations associated with a neurological, musculoskeletal or developmental disorder [77]. If a child’s cognitive challenges limit the quality of interview data, we will rely on the parent interview as the primary data source. Data will be gathered with semi-structured interviews. Questions asked of children and parents will be similar, however questions will be modified in the case of children due to age and cognitive ability. Families will be asked about their experiences with and recommendations for improving health services to manage SO in children with disabilities. Interview data will be digitally recorded, transcribed verbatim, and subsequently managed using *NVivo 10* (QSR International). Demographic and clinical data will be collected for descriptive purposes. Guided by an ecological perspective [74], we will use thematic data analysis [78] to identify family experiences and recommendations at the family, social and health care services levels.

### Outcomes

This qualitative study will reveal families’ experiences and recommendations for improving health services to manage SO in children with disabilities, inform modifications to health services delivery for managing obesity in children with disabilities, and identify intervention approaches to best meet the needs of this population.

#### Study 5: Examining obesity-related health outcomes using the 4Ms framework (metabolic, mechanical, mental, milieu) in children with SO

### Rationale and objectives

Most studies linking SO in children to adverse health outcomes risk such as type 2 diabetes and cardiovascular disease have focused on cardiometabolic health (e.g., insulin resistance, hypertension, dysglycemia, dyslipidemia) [8, 20, 21]. However, biomechanical, psychological and social health measures have received much less research attention, yet may be most salient for families and clinicians [79]. Further, the lack of universally-accepted criteria for SO in children highlights the need for empirical evidence to inform a definition. We aim to identify the presence of adverse health outcomes, namely metabolic, mechanical, mental health, and social milieu (the 4Ms), and compare and contrast 4Ms in children across the range of obesity and across definitions of SO.

### Research plan

Using cross-sectional baseline data collected from children and families enrolled in CANPWR [55], we will examine the burden of illness based on a diverse set of conditions organized under the 4M framework. Health measures under the 4Ms framework are (1) metabolic (fasting levels of glucose, insulin, total cholesterol, HDL-cholesterol, LDL-cholesterol, triglycerides, systolic and diastolic blood pressure, liver enzymes, Acanthosis Nigricans); (2) mechanical (sleep quality and apnea, musculoskeletal problems, gastroesophageal reflux disorder, physical functioning); (3) mental health (depression, anxiety, attention deficit hyperactivity disorder, learning disability, emotional functioning); and (4) social milieu (household income, parent education and health status, school and social functioning, inter-personal interactions [e.g., bullying]). Our secondary data analysis will utilize data already collected within CANPWR using validated questionnaires that are standardized across sites. We will compare and contrast variables across the 4M categories in a sample of CANPWR participants (n = 1600) along a spectrum of increased weight status: overweight (BMI ≥85th percentile), obese class I (BMI ≥95th percentile), obese class II (BMI ≥120% of the 95th percentile), and obese class III (BMI ≥140% of the 95th percentile). This classification system was proposed recently [19, 80], but lacked empirical data. Consideration will also be given to BMI ≥99th percentile. Descriptive statistics will be calculated for participant characteristics and health outcomes (e.g., means, 95% CIs, proportions), for continuous and categorical data. Group differences will be examined by analysis of (co)variance with post-hoc comparisons and Chi squared tests for continuous and categorical data, respectively.

### Outcomes

This study will examine a diverse set of health outcomes associated with obesity across a spectrum of excess weight for children enrolled in CANPWR, contribute data to determine criteria for SO based on health risks, and quantify the burden of illness associated with SO in children (as in adults [81, 82]) to inform development of clinical tools and decision-making.

#### Study 6: Does integration of health coaches improve adherence to an e-health lifestyle and behavioral intervention? A randomized controlled trial (RCT) of LiGHT (Living Green and Healthy for Teens)

### Rationale and objective

Geographic or physical barriers prevent access to in person health services for some children with SO, while others prefer self-guided treatment options. E-health strategies offer a cost-effective means to broadly disseminate lifestyle and behavioral interventions through the Internet. However, the extent to which participants adhere to web-based intervention components remains relatively low [83], limiting intervention effects [83–88]. LiGHT (Living Green and Healthy for Teens) is an e-health lifestyle and behavioral intervention for managing SO in children. Previously, families rated LiGHT favorably and expressed a desire to incorporate health coaches into LiGHT to enhance engagement, support and motivation [89]. We will examine whether adding health coaches to LiGHT increases intervention adherences and improves anthropometry, lifestyle habits, cardiometabolic risk factors, and family psychosocial health.

### Research plan

We will conduct a seven-site comparative effectiveness RCT with two parallel groups and a 1:1 allocation ratio across Canada. Our protocol adheres to the Standard Protocol Items for Randomized Trials guidelines [90] and will be registered publically and prospectively at ClinicalTrials.gov.

#### Intervention groups

LiGHT is an e-health intervention designed for children and their families, delivered in 12 modules. It combines evidence-based obesity management techniques with environmental and economic information, and is made available to families through a secure, password-protected website via desktop, laptop or tablet. LiGHT is designed to increase intervention adherence and retention by emphasizing the impact on personal health outcomes of individual lifestyle and behavioral habits, the environment (e.g., food packaging materials) and family finances (e.g., commuting costs). Following feedback from the pilot study [91], we are incorporating gamification into LiGHT (*v*2.0) to enhance visual appeal and interactivity. For this trial, we will compare LiGHT (*v*2.0) to LiGHT+, which adds personal health coaches (PHCs) to improve intervention adherence instead of providing a virtual coach. PHCs will encourage LiGHT+ participants to complete behavioral techniques (e.g., self-monitor, set goals) that can enable changes in eating and physical activity habits [91]. They will support families during the trial through their preferred mode of contact (text message, email and/or telephone). PHCs will have health professional training (e.g., psychology, nutrition), and be employed locally at each site as research assistants. Team members with expertise in health coaching and communication will train PHCs at study onset and support them throughout the trial. Children are the primary intervention recipients, with parents playing secondary roles. Children and parents in both trial groups will complete a comprehensive assessment at 0-, and 4-months follow-up.

#### Participants

Children 10–17 years old with SO (BMI ≥99th percentile) [17] and at least one parent (primary caregiver) will be eligible to participate. Families will be recruited through six CANPWR sites across Canada. In our team members’ experience, ~50% of families attending an information session do not initiate care. As an alternative to in-person care, we will recruit families who decided not to initiate care after initial referral to one of the seven sites, a strategy that offers an alternative for families who declined in-person care and avoids co-intervention effects with families currently enrolled in a clinical program.

#### Outcome measures

Our primary outcome will be adherence. This outcome will be tracked continuously within LiGHT and measured as a latent variable by monitoring the extent to which participants access the intervention (number of weeks accessed), percentage of pages viewed, and adherence to behavioral change techniques (percentage of use of self-monitoring techniques). Secondary outcomes include children’s anthropometry, lifestyle habits, cardiometabolic health measures, family psychosocial health, and factors at individual, social and environmental levels that can influence adherence (e.g., intrinsic motivation, peer support). All outcomes will be measured at 0-, and 4-months follow-up using standardized measures.

#### Sample size

We will recruit 186 participants in two groups (n = 93/group). The trial can detect a 20% difference in adherence (odds ratio of 2.33) at 4-months follow-up at an alpha of 0.05 and 80% power.

#### Randomization and blinding

A biostatistician will complete the computer-generated random allocation sequence, in blocks of variable sizes. Allocation concealment will be achieved by a central randomization system. Individuals collecting families’ outcome data and completing data analysis will be blind to group allocation, which will be concealed until data analyses are completed.

#### Data management and analysis

We will capitalize on technical and research support from the University of Alberta Women and Children’s Health Research Institute to use REDCap, a secure online platform, for data management and storage. Baseline characteristics and outcomes will be calculated with appropriate descriptive statistics (e.g., means, 95% CIs, proportions). LiGHT has built-in capacity to gather metrics on intervention use and adherence. To examine our primary objective, we will compare the difference in adherence between LiGHT and LiGHT+ using a risk difference estimator based on logistic regression [92]. We will also use generalized estimating equations to model change in adherence over time to identify individual, social and environmental variables associated with adherence. Adherence will be modeled using an auto-regressive correlation structure to account for the expectation that adherence will decrease over time. To evaluate our secondary objective, we will compare groups using the same risk difference estimator procedure.

### Outcomes

This trial will determine whether health coaching enhances adherence (and other health-related outcomes) to LiGHT and provide further evidence for this novel and accessible treatment option for managing SO in children who are referred for, but decline, in-person care.

#### Study 7: STOMP early years: a pilot randomized controlled trial of an intensive, family-centered, home visiting intervention for young children with SO

### Rationale and objectives

To date, few reports have been published on obesity management interventions in young children (<5 years old) [30]. Evidence is emerging that community—[93] and home-based [94, 95] interventions can help young children to improve their health behaviours and weight status. Interventions available to families in their local communities and homes can reduce barriers to accessing health services [96]. The SickKids Team Obesity Management Program (STOMP) Early Years Program is a unique and intensive pediatric obesity management program designed for 1 to 5 year olds with SO and their families. This evidence-based program combines lifestyle, behavioral and parenting strategies and is offered in partnership with Toronto-based public health professionals with a mandate to provide both community- and home-based care. In our health services experience to date, the intervention is acceptable to families and health care providers, but the feasibility of scientific issues (e.g., sample size, feasibility of family consent and outcome measurement tools) remains unknown. Overall, we will determine the feasibility of using this intervention to manage SO in young children, obtain a reliable estimate of the variance in the primary outcome (BMI z-score) and use data from this study to calculate a sample size estimate for a definitive, future RCT.

### Research plan

We will conduct a single-site (Toronto) internal pilot RCT with two parallel groups and a 1:1 allocation ratio. This internal pilot RCT [97, 98] will be co-led in partnership with Toronto Public Health. Our protocol adheres to the Standard Protocol Items for Randomized Trials guidelines [90] and will be registered publically and prospectively at ClinicalTrials.gov.

#### Intervention and control groups

Parents randomized to the intervention group will participate in a modified, version of the Chicago Parenting Program [99] (10 weekly sessions over 6 months), which focuses on lifestyle, behavioral and parenting issues and strategies for managing SO in young children. A mental health specialist–dietitian–nurse team will deliver the first four sessions, which focus on health behaviours. The next 6 sessions will be delivered by a public health nurse and focus on parenting skills to help implement behaviour change. This is complemented by four home-based visits by a public health nurse. Home visits help families to incorporate healthy nutrition and physical activity habits into their home environments through effective parenting practices learned in the program. Families randomized to the control group will be offered this program as a delayed treatment following the study period. Children and parents in both groups will complete a comprehensive multidisciplinary assessment at 0- and 6-months follow-up. To minimize the risk of bias, study data will be collected by a trained research assistant who will not participate in intervention delivery.

#### Participants

Young children (1–5 years old) with SO (BMI ≥99th percentile) and at least one parent (primary caregiver) who are referred for the STOMP program will be eligible to participate. Families will be excluded if they reside beyond the Toronto Public Health catchment area for home visiting.

#### Outcome measures

Our primary outcome will be BMI z-score. Secondary outcomes will include children’s dietary intake (e.g., NutriSTEP^®^ [100]), physical activity, sedentary behavior (e.g., Nutrition and Health Questionnaire [NHQ]–questions based on the Canadian health Measures Survey [CHMS] [101]), cardiometabolic risk factors (e.g., blood pressure, lipids, insulin resistance), and family psychosocial health (e.g., Parental Stress Index [102]).

#### Sample size

Recommendations for an *internal pilot study* are to include half the anticipated sample size for a full-scale trial and at least 10 children/group [97]. We will include a total of 38 children (n = 19/group) for this pilot RCT. A power calculation is not appropriate as the study does not aim to provide a definitive estimate of treatment effect. The aim is to provide robust estimates of the likely rates of recruitment and retention, and to yield estimates of the variability of the primary and secondary outcomes to inform power calculations for a future large-scale trial [103, 104].

#### Randomization and blinding

A biostatistician will complete the computer-generated random allocation sequence, in blocks of variable sizes. Allocation concealment will be achieved by a central randomization system. Individuals collecting families’ outcome data and completing data analysis will be blind to group allocation, which will be concealed until data analyses are completed.

#### Data management and analysis

The Applied Health Research Centre (University of Toronto) will provide support for data management and analysis. Baseline characteristics and outcomes will be calculated with appropriate descriptive statistics (e.g., means, 95% CIs). Feasibility will be assessed as our ability to recruit, consent and collect data from families at 0- and 6-months follow-up. We will calculate variance around the primary outcome (BMI z-score) in the control group to inform sample size for a definitive future RCT.

### Outcomes

This study will provide essential data and experience to plan a definitive, multi-site RCT for managing SO in young children [90] and enable our team to partner with public health professionals (both front-line staff and decision-makers) to manage SO in the community. This trial will offer invaluable experience to evaluate the STOMP Early Years program in other communities.

## Discussion

The aforementioned studies represent collaborative research that will be conducted in a decentralized manner. Our project leaders are based at 9 institutions across 5 Canadian provinces, an organizational reality that highlights our geographic diversity, but necessitates defined structures and functions (described below) that enable effective and timely interactions between team members so that our research activities are optimized.

### Team organization and communication

**Fig. 2:**
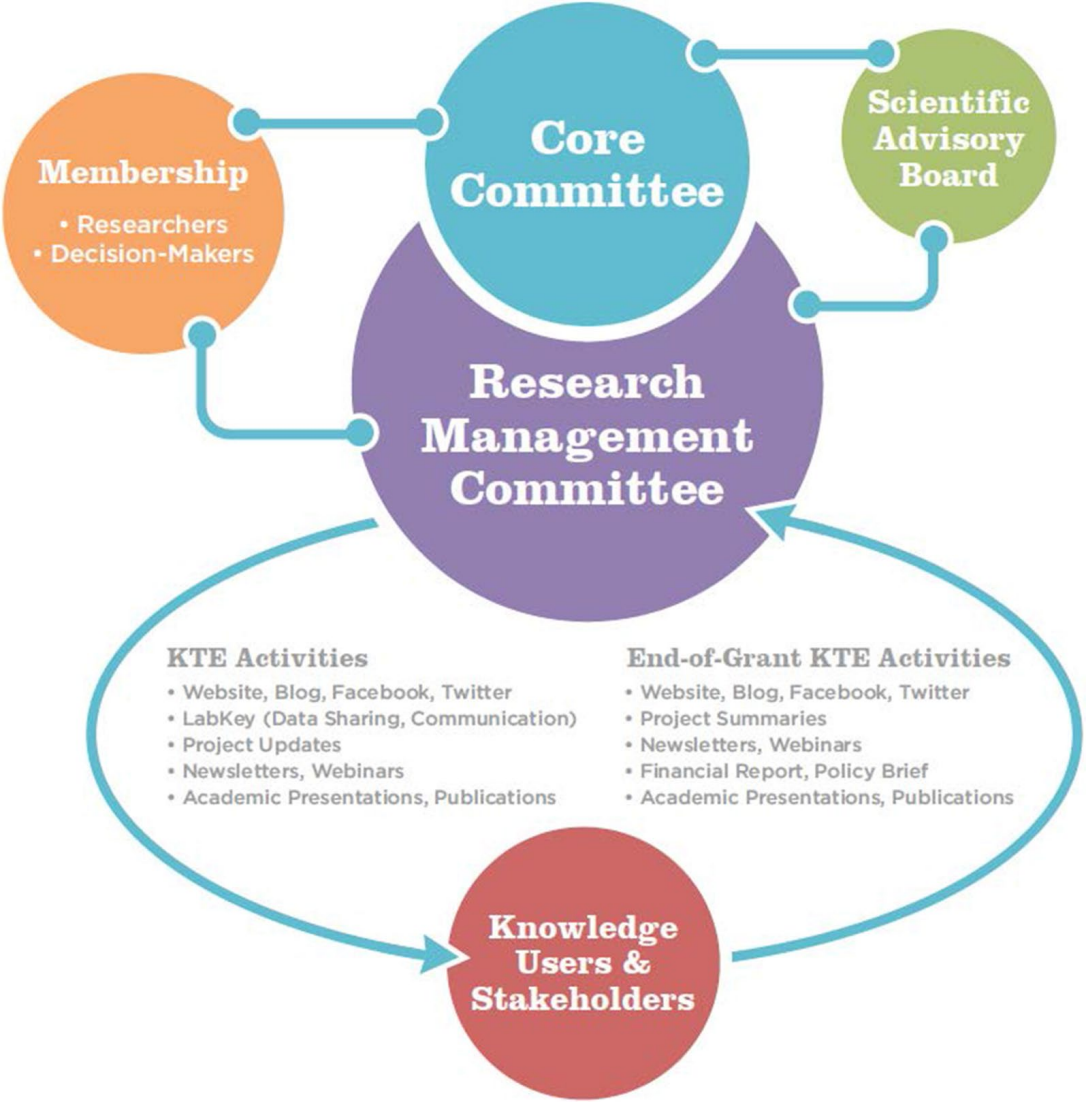
Team governance model interactions and knowledge translation and exchange activities with knowledge users and stakeholders

Our team consists of emerging and established researchers and trainees, a diverse group of decision-makers (DMs) that represent a mix of provincial and national portfolios, and a Scientific Advisory Board (SAB) consisting of national and international leaders with expertise in SO, pediatric medicine, psychology, primary care, health care policy and public health, as well as research and administrative leadership and advocacy. Drawing on elements of successful research-to-practice networks, our network governance structure (Fig. 2) will emphasize frequent exchanges among all team members, interactions that are essential to create and sustain a successful research-to-practice network [105]. This model will allow us to reach outcomes that could not be realized by team members individually, and to apply best practices in managing research studies, monitoring progress and optimizing collaborations between team members.

### Core Committee

The Core Committee (CC) will lead our team and assumes final responsibility for all activities. They will monitor our team’s budget, timelines and deliverables. Members include all principal investigators, two DMs, and three emerging researchers. All decisions will be made by consensus. The CC will meet quarterly by teleconference and advise the Research Management Committee.

### Research Management Committee

The Research Management Committee (RMC) will include all researchers and DMs. They will organize our scientific agenda, plan and implement Knowledge Translation and Exchange (KTE) activities, facilitate training and educational opportunities, develop and administer policies on authorship and dispute resolution. Working groups will form out of the RMC with varied foci (e.g., KTE; training and education), presenting opportunities for different team members to assume leadership roles. All RMC decisions will be made by consensus; meetings will be held every 2 months by teleconference.

### Annual general meeting (AGM)

Our AGM will be held annually and all team members (researchers, DMs, SAB) will be encouraged to attend. Our SAB members will have opportunities to interact with team researchers and DMs throughout the year, but the AGM will provide valuable formal and in-person interactions. AGMs will be planned as satellites to existing national obesity conferences attended regularly by most researcher team members. Participation for those not attending an AGM will be enabled by teleconference.

### Training and mentoring plan

We aim to build skills and experience for graduate students, fellows, and new investigators to give them the competence and network connections for effective health services research in obesity. All planned studies include at least one graduate student, fellow, or new investigator. Our team will capitalize on established programs and infrastructure. For instance, the Canadian Obesity Network (CON) will be an important training partner. Student and New Professional (SNP) chapters of CON are in place at all team universities, with a focus on networking and continuing education. The CON-SNP mandate matches our training goals precisely, to increase the number of future clinicians and academics networking and learning through in-person and virtual means to help prevent and manage obesity in Canada. As part of our AGMs, our team will plan and provide brief interactive workshops delivered by both team members and outside experts on topics of special relevance to our research program (e.g., designing clinical care pathways, reviewing ethical considerations in health services to manage SO in families with multiple stresses). All team members will be invited to participate in these workshops, but our emerging team members will be strongly encouraged to attend.

### Integrated Knowledge Translation And Exchange plan

**Fig. 3:**
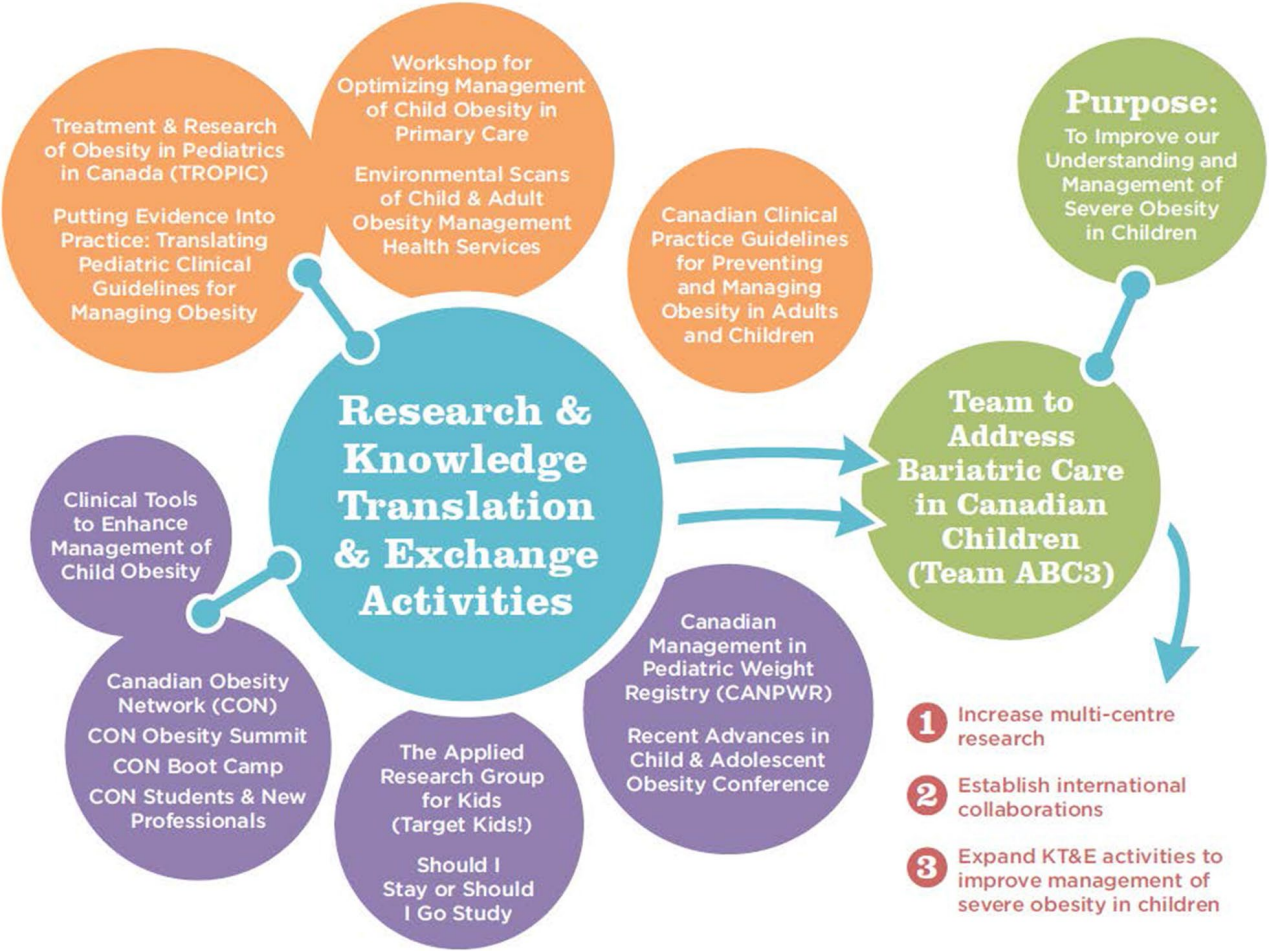
Past, ongoing, proposed, and future research and knowledge translation and exchange activities related to managing pediatric obesity in Canada

Our KTE strategies (Fig. 3) optimize existing, genuine relationships and include tailored messages to each KTE partner [106–109]. All team members will be encouraged to contribute to multiple KTE activities (e.g., blog, social media, webinars, policy briefs, manuscripts), capitalizing on our diverse health services and organizational perspectives. Our RMC will be the primary venue for information exchange between team members. To connect with researchers and clinicians, we will work with our team members (DMs in particular) to identify timely and appropriate settings for sharing study findings with end-user audiences. Examples include plain-language summaries of study updates for dissemination within local research institutes (e.g., Child and Family Research Institute, University of British Columbia) and health care organizations. Our provincially-placed DMs representing Alberta Health Services and Ontario’s Ministry of Health and Long-Term Care will provide us with venues to share our findings through traditional and contemporary options to targeted audiences within their organizations. DMs affiliated with national organizations such as Dietitians of Canada and the Canadian Foundation for Dietetic Research include both clinicians and scientists. We will share our research with these two groups of nutrition professionals through continuing education events. Based on team members’ experience in integrated KTE, Team ABC3 assembled a Social Media Subcommittee (SMS) that will continue post-funding. Our social media plan is intended to keep team members and interested stakeholders up to date on our team’s research activities and disseminate research, academic and clinical information about pediatric obesity (in general) and severe pediatric obesity (in particular). Our plan is currently being implemented through our team blog (http://www.teamabc3.wordpress.ca) and twitter (http://www.twitter.com/team_abc3) to promote dialogue, transparency, awareness, and reputation. Our SMS members include two researchers, one DM, our research coordinator and an external engagement and communications professional.

### Monitoring and evaluation

To evaluate our team’s performance, we will apply the Transdisciplinary Tobacco Use Research Centres survey to evaluate team collaborative processes and transdisciplinary integration [110]. We will administer this survey at our first AGM to generate baseline data, and annually thereafter to assess team members’ perception and experience of collaborative, transdisciplinary team interactions. Anonymized results will be shared within the team to identify opportunities to enhance our work and to celebrate achievements. To determine the impact and reach of our web-based content, we will use Google Analytics (e.g., number of page views, length of stay on website, number of document downloads) and Twitter Analytics (e.g., number of retweets and likes, engagement rate, reach of tweets).

### End-of-grant knowledge translation

We will use prevailing dissemination methods to reach researchers and clinicians. Principal Investigators will attend Canadian academic obesity conferences to share information on team progress and results. Team members will further share study findings at international meetings (e.g., The Obesity Society, International Congress on Obesity) to expand professional networks in the obesity research community and disseminate our research outputs broadly. Our studies will generate peer-reviewed publications, including at least ten primary publications and numerous secondary manuscripts. To reach decision-makers, a final report of findings from our multiple studies will follow a formula from the Canadian Foundation for Healthcare Improvement. This plain-language report will communicate study findings and implications to a diverse group of provincial and national stakeholders. We will publish it through our social media channels and link it with team members’ affiliated organizations. A policy brief with recommendations on incorporating key findings into clinical practice will accompany the final report to key policymaking associations (e.g., Dietitians of Canada, Canadian Paediatric Society). We will draw on the extensive experience of our researchers and DMs in authoring reports and policy briefs for stakeholders.

## Conclusions

High quality evidence is urgently needed for children with SO in order to optimize care and delivery of health services. The diversity of initiatives in our research aligns with national priorities in obesity research; brings together leading scientists, clinicians, and stakeholders from across the country; and will inform health services delivery in Canada to provide the best care possible for our children with SO and their families. Overall, this research is essential to produce and translate the high quality evidence needed for this vulnerable and at-risk population.

## Abbreviations

4Ms: metabolic, mechanical, mental health, and social milieu
AGM: annual general meeting
BMI: body mass index
BORN: Better Outcomes Registry and Network (Ontario)
CANPWR: Canadian Pediatric Weight management Registry
CC: Core Committee
CIHR: Canadian Institutes for Health Research
CON: Canadian Obesity Network
DMs: decision-makers
ED: emergency department
EMERALD: Electronic Medical Record Administrative Data Linked Database
ICES: Institute of Clinical Evaluative Sciences
KTE: Knowledge Translation and Exchange
LiGHT: Living Green and Healthy for Teens
LPA: Latent Profile Analysis
OHIP: Ontario Health Insurance Plan
PHCs: personal health coaches
RCT: randomized controlled trial
RMC: Research Management Committee
SAB: Scientific Advisory Board
SMS: Social Media Subcommittee
SNP: Student and New Professional
SO: severe obesity
STOMP: SickKids Team Obesity Management Program
TARGet Kids!: The Applied Research Group for Kids
Team ABC3: Team to Address Bariatric Care in Canadian Children
